# Manganese Superoxide Dismutase and Breast Cancer Recurrence: A Danish Clinical Registry-Based Case-Control Study, and a Meta-Analysis

**DOI:** 10.1371/journal.pone.0087450

**Published:** 2014-01-31

**Authors:** Deirdre P. Cronin-Fenton, Mariann Christensen, Timothy L. Lash, Thomas P. Ahern, Lars Pedersen, Jens Peter Garne, Marianne Ewertz, Herman Autrup, Henrik T. Sørensen, Stephen Hamilton-Dutoit

**Affiliations:** 1 Department of Clinical Epidemiology, Aarhus University, Aarhus, Denmark; 2 Institute of Pathology, Aarhus University, Aarhus, Denmark; 3 Department of Epidemiology, Rollins School of Public Health, Emory University, Atlanta, Georgia, United States of America; 4 Department of Surgery, University of Vermont College of Medicine, Burlington, Vermont, United States of America; 5 Danish Breast Cancer Cooperative Group, Copenhagen, Denmark; 6 Department of Breast Surgery, Aalborg University Hospital, Aalborg, Denmark; 7 Department of Oncology, Odense University Hospital, Institute of Clinical Research, University of Southern Denmark, Odense, Denmark; 8 Institute of Public Health, Aarhus University, Aarhus, Denmark; Sanjay Gandhi Medical Institute, India

## Abstract

**Background:**

Manganese superoxide dismutase (MnSOD) inhibits oxidative damage and cancer therapy effectiveness. A polymorphism in its encoding gene (*SOD2: Val16Ala* rs4880) may confer poorer breast cancer survival, but data are inconsistent. We examined the association of *SOD2* genotype and breast cancer recurrence (BCR) among patients treated with cyclophosphamide-based chemotherapy (Cyclo). We compared our findings with published studies using meta-analyses.

**Methods:**

We conducted a population-based case-control study of BCR among women in Jutland, Denmark. Subjects were diagnosed with non-metastatic breast cancer from 1990–2001, received adjuvant Cyclo, and were registered in the Danish Breast Cancer Cooperative Group. We identified 118 patients with BCR and 213 matched breast cancer controls. We genotyped *SOD2* and used conditional logistic regression to compute the odds ratio (OR) and associated 95% confidence intervals (95% CI) of BCR. We used random-effects meta-analytic models to evaluate the association of *SOD2* polymorphisms and BCR.

**Results:**

The frequency of the *SOD2-Ala* allele was 70% in cases versus 71% in controls; 40% versus 44% were heterozygotes, and 30% versus 25% were homozygotes, respectively. Heterozygote and homozygote carriers of the *Ala* allele had no increased rate of BCR (OR = 1.1, 95%CI = 0.65, 2.0, and OR = 0.87, 95%CI = 0.47, 1.6, respectively). Five studies informed the meta-analytic models; summary estimates associating BCR for homozygote, or any inheritance of the variant *Ala* allele were 1.18 (95%CI = 0.74, 1.88), and 1.18, (95%CI = 0.91, 1.54), respectively.

**Conclusion:**

Our findings do not suggest that MnSOD enzymatic activity, as measured by *SOD2* genotype, affects rates of BCR among patients treated with Cyclo.

## Introduction

Manganese superoxide dismutase (MnSOD) plays a crucial role in endogenous defense mechanisms against reactive oxygen species (ROS) by catalyzing their conversion into H_2_O_2_ and O_2_ in the mitochondria. MnSOD is encoded by the superoxide dismutase (*SOD2*) gene (locus 6q25.3). A single nucleotide polymorphism (SNP) in the *SOD2* gene (a T to C substitution) results in a valine (Val) to alanine (Ala) amino acid change, and higher MnSOD enzymatic activity [1].

Chemotherapy exerts its anti-cancer effects in part, by inducing the production of ROS. Some studies suggest that the *SOD2* polymorphism acts as a predictor of response to chemotherapy in cancer patients [2]–[5]. Research has also suggested that the Ala/Ala genotype may be associated with an approximately two-fold increase in risk of breast cancer [6]–[8], but that the Val/Val genotype may correlate with tumors with higher metastatic potential (lymph node positive disease) [9]. Its association with breast cancer recurrence is not clear because of conflicting findings from the seven former studies [10]–[15]. Two large studies (>500 patients) reported that breast cancer patients with the Ala allele had poorer survival compared with carriers of the Val allele [10], [11]. In contrast, smaller studies (n∼300 patients) reported better survival among carriers of the Ala allele, especially in patients treated with radiation in addition to chemotherapy [13], [14], [16]. Studies finding no association between MnSOD activity and breast cancer recurrence have also been reported [15], [17]. Researchers have suggested that findings from the individual studies should be assembled in a meta-analysis in order to better define the association of SOD2 polymorphisms and outcomes in patients with breast cancer [14].

In addition to their conflicting results, the previous studies have had limited generalizability due to the inclusion of selected patient groups such as those with metastatic disease [18], or hormone receptor-positive tumors [11]. Studies have lacked information on other cancer-directed therapies [10], such as radiation therapy, whose effectiveness has been shown to be modified by *SOD2* polymorphisms [13], [19]. Cyclophosphamide remains a standard chemotherapy for the treatment of breast cancer patients and since 2010 has been used in combination with epirubicin and docetaxol [20]. Therefore, we conducted a population-based case-control study among women treated with cyclophosphamide-based chemotherapy (cyclophosphamide-epirubicin-5-fluorouracil – CEF) in Denmark to evaluate the effect of the *SOD2* polymorphism on breast cancer recurrence. We used meta-analytic techniques to compare our findings with published studies on the association of SOD2 and breast cancer survival.

## Methods

Patient consent was not required. The study was approved by the Regional Committee on Biomedical Ethics of the Central Denmark Region (20070085/1-10-72-614-12), the Danish Data Protection Agency (2012-41-1399), and the Danish Breast Cancer Cooperative Group. These permissions waived the need for written informed consent from the participants.

### Study Population and Data Collection

The source population was female residents of Denmark’s Jutland peninsula, aged 35–69 years, diagnosed between 1990 and 2001 with stage I-III breast cancer and registered with the Danish Breast Cancer Cooperative Group (DBCG). Each patient had received adjuvant CEF primary chemotherapy, with or without other adjuvant treatments. Follow-up time began one year after the breast cancer diagnosis date and continued until the date of the first breast cancer recurrence, death from any cause, loss to follow-up (*e.g.,* emigration), ten years of follow-up, or September 1, 2008.

Breast cancer patients registered in the DBCG are routinely followed-up twice yearly for the first five years after initial diagnosis, and then annually thereafter for a further five years. Cases were women in the source population diagnosed with a local or distant breast cancer recurrence during follow-up. The DBCG defines breast cancer recurrence as a recurrence in the same breast, a new primary tumor in the ipsi- or contra-lateral breast, or a recurrence at a site other than the original primary cancer site [21].

**Figure 1:**
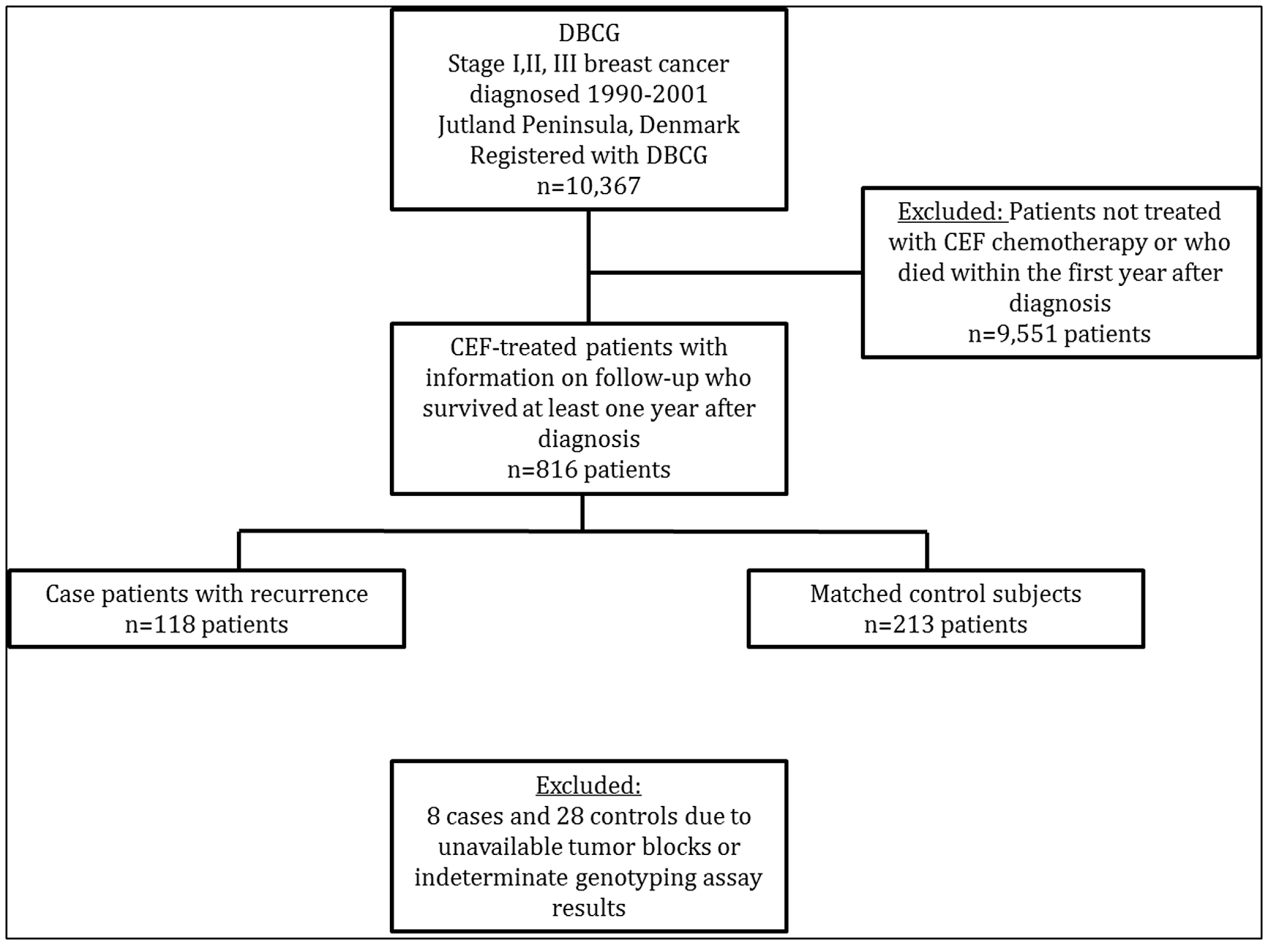
Design used to identify the study sample. The source population consisted of 10,367 female residents of the Jutland Peninsula in Denmark aged 35–69 years who were diagnosed with stage I, II, or III breast cancer between 1990 and 2001. Most of the women were excluded because of unknown protocol or death within the first year after diagnosis (n = 9,551). Genotyping results were missing for a small proportion of patients due to unavailable tumor blocks or indeterminate assay results.

For each case, we selected controls using risk-set sampling without replacement from members of the source population. Controls were women who received CEF as their primary treatment, who survived at least one year, and who had not had a breast cancer recurrence at the time their corresponding case experienced a recurrence – risk-set sampling [22]. We individually matched up to two breast cancer controls to each case on birth year (+/−1 year), county of residence, cancer stage, estrogen receptor status, and date of diagnosis (calliper matched +/−1 year). Figure 1 shows an overview of the breast cancer patients included in this study.

### Tissue Processing, DNA Extraction, Amplification, and Genotyping

We retrieved the formalin-fixed, paraffin-embedded (FFPE) primary tumors of cases and controls from the pathology archives of treating hospitals [23]. We reviewed hematoxylin and eosin stained sections and pathology reports to identify appropriate tumor blocks for processing. All tissue blocks were processed and handled using standard operating procedures to minimize risks of cross-contamination and nuclease digestion. Three to six 10 µm sections were cut from each tumor block and placed in sterile 1.5 mL centrifuge tube for DNA extraction.

After xylene deparaffinization, DNA was extracted by two incubations in 99% ethanol, proteinase K digestion, and purification with the QIAamp DNA FFPE Tissue Kit (Qiagen AB, Dusseldorf, Germany) following the manufacturer’s protocol.

Fifty nanograms of purified DNA from each tumor sample were amplified in 25 uL PCR reactions: 95°C for 10 minutes, followed by 40 cycles of 92°C for 15 seconds, and 60°C for 60 seconds, using primers and reagents supplied with the TaqMan genotyping kit (Applied Biosystems, Foster City, California, USA).

A commercially available TaqMan kit was used to genotype *SOD2* (rs4880) (Applied Biosystems assay ID C_8709053). All samples were assayed in duplicate using the MX3000P Real-Time PCR system (Stratagene, Cedar Creek, Texas, USA). Positive genotyping controls for the variant alleles were identified by sequencing lymphocyte DNA from discarded and anonymized blood samples of 30 healthy donors, and included with each batch of assays. Negative controls, with sterile water substituted for DNA, were also included in each batch. Laboratory personnel were blinded to all clinical data, including case/control status.

### Definitions of Analytic Variables

We classified *SOD2* genotype as homozygous wildtype (Val/Val alleles), heterozygous (Val/Ala allele), and homozygous variants (Ala/Ala alleles), according to the auto-call feature of the analytic software (MXPro QPCR version 4.1, Stratagene). We additionally classified the *SOD2* genotype as inheritance of any Ala allele (Val/Ala and Ala/Ala) versus homozygous wildtype (Val/Val).

Covariates included the year of breast cancer diagnosis, age at diagnosis, menopausal status at diagnosis (premenopausal versus postmenopausal), type of breast cancer primary surgery (mastectomy or breast conserving surgery), receipt of adjuvant tamoxifen, and receipt of adjuvant radiation therapy.

### Statistical Analyses

We computed the frequency of cases and controls within each category of all analytic variables. We used conditional logistic regression to compute the odds ratio (OR) and associated 95% confidence interval (CI) (as a measure of the recurrence rate ratio) to analyse the association of the *SOD2* genotype with the rate of breast cancer recurrence. Because of the matched design, the conditional OR was adjusted for confounding by the matching factors (birth year, stage, county, ER status and date of diagnosis), menopausal status, primary surgery type, and receipt of radiation therapy. We tested whether genotypes at the *SOD2* locus observed among control subjects were in Hardy-Weinberg equilibrium by computing the χ^2^ test statistic, with expected genotype frequencies based on the observed prevalence of major and minor alleles [24].

### Meta-analysis

#### Search strategy

We searched for the terms “manganese superoxide dismutase,” “MnSOD,” “SOD2,” “breast cancer” and “survival” in PubMed. No language restrictions were imposed. All papers published through November 2013 regarding the association between *SOD2* gene variants and risks of breast cancer recurrence or mortality were reviewed to determine whether their results should be included.

#### Data sources and searches

We searched for the terms “manganese superoxide dismutase,” “breast cancer” and “survival” in PubMed, and imposed no language restrictions. We also searched references of the included articles.

#### Study selection

Our search targeted articles that met the following criteria: evaluated a prognostic outcome (breast cancer recurrence, mortality, or breast cancer survival) in breast cancer patients by MnSOD expression, evaluated original data, and reported a risk estimate with an associated estimate of precision (standard error or 95%CI). We included studies that genotyped *SOD2* in breast cancer patients, and correlated it with breast cancer prognosis (breast cancer recurrence, mortality or breast cancer survival) in patients treated with cyclophosphamide-based chemotherapy. We excluded studies where *SOD2* had been correlated with cancer therapies other than chemotherapy.

#### Data extraction

DCF performed the PubMed searches, reviewed each of the retrieved abstracts, selected those that appeared to meet the study’s inclusion criteria, and reviewed each of the selected manuscripts in full. Where a study met the inclusion criteria, DCF extracted the data from each study (effect estimates and associated 95% confidence intervals), of the included manuscripts, and performed the meta-analysis. All authors reviewed and agreed on the study inclusion criteria, and on the selected manuscripts. Where there was a disagreement, the paper was re-reviewed and an agreement was reached.

#### Extracted data variables

We retrieved information on several breast cancer prognostic outcomes namely, breast cancer mortality, breast cancer recurrence, disease-free survival, and breast cancer-specific mortality.

Given the differences in study design, we expected substantial heterogeneity between the studies. We formally assessed this by using Cochran Q and I^2^ [25] and observed substantial heterogeneity (Q, 12.74 on 4 *df*, P = 0.013; I^2^, 68.6%, P = 0.02). We calculated a pooled effect estimate using the Der Simonian-Laird methods for random-effects models. [26].

#### Bias assessment

We evaluated publication bias by constructing a funnel plot (Figures A & B in File S1). We also performed Duval and Tweedie nonparametric “trim and fill” procedure to further investigate any potential effects of publication bias. This method creates hypothetical missing studies, imputes their effect estimates, and recalculates a pooled effect estimate [27].

#### Meta-analytic statistical models

We created two separate meta-analytic models to investigate the gene-dose effect of *SOD2* alleles and breast cancer outcomes. The first, a recessive model, evaluated recurrence risks associated with inheritance of two variant alleles (Ala/Ala) compared with homozygous or heterozygous carriers of the wild-type allele (Val/Val and Val/Ala, respectively). The second, a dominant model, evaluated recurrence risks associated with inheritance of any Ala allele (Ala/Ala or Val/Ala), compared with homozygous carriers of the wild-type allele. For the second model, where studies presented associations for heterozygote and homozygote variant alleles separately, we estimated an inverse-variance-weighted average of these two associations and used this estimate in the model.

All analyses were performed using STATA software, version 11.0 (StataCorp LP, College Station, Texas). All statistical tests were two-sided.

## Results

We identified 118 patients with recurrent breast cancer and 213 matched controls. Table 1 provides descriptive characteristics of the study population. Because of the matching, the proportions of cases and controls by age group, stage and ER status were similar. More cases than controls had a mastectomy as their primary surgical treatment (84% versus 76%). A higher proportion of cases than controls received CEF alone rather than in combination with pamidronate (PAM), tamoxifen (TAM), or herceptin (HER).

**Table 1:** Frequency of patients with breast cancer recurrence (n = 118) and matched controls (n = 213), by clinical characteristics, among women aged 35 to 69 years at the time of breast cancer diagnosis who resided in Jutland, Denmark, 1991–2001.

Category	Subcategory	Cases (n,%)	Controls (n,%)
**Age at diagnosis**			
	35–44	49 (42)	84 (39)
	45–54	55 (47)	106 (50)
	55–64	8 (6.8)	18 (8.5)
	65–69	6 (5.1)	5 (2.3)
**Menopausal status**			
	Premenopausal	95 (81)	187 (88)
	Postmenopausal	23 (19)	26 (12)
**Stage**			
	I	11 (9.3)	25 (12)
	II	49 (42)	86 (40)
	III	58 (49)	102 (48)
**Estrogen receptor status**			
	Poorˆ	72 (61)	119 (56)
	Positive	46 (39)	94 (44)
**Surgery type**			
	Mastectomy	99 (84)	162 (76)
	Breast conserving surgery	19 (16)	51 (24)
**Radiation therapy**			
	No	35 (30)	61 (29)
	Yes	83 (70)	152 (71)
**Adjuvant therapy**			
	CEF	74 (63)	113 (53)
	CEF+PAM*	12 (10)	22 (10)
	CEF+TAM^§^	31 (26)	78 (37)
	CEF+TAM+HER^¤^	1 (0.9)	0
**DBCG Protocol**			
	DBCG89	55 (47)	86 (40)
	DBCG99	63 (53)	127 (60)

* PAM =  pamidronate; ^§^TAM =  tamoxifen; ^¤^HER =  Herceptin; ?ER ”poor” refers to <10% ER positivity as per the diagnostic period of patients included in the current study.

MnSOD genotypes were in Hardy-Weinberg equilibrium among the controls (P = 0.07) (Table 2). Table 3 presents the association between *SOD2* genotype and breast cancer recurrence. Forty percent of cases versus 44% of controls were heterozygotes (Val/Ala), while 30% of cases and 25% of controls were homozygote variants (Ala/Ala). In both conditional and adjusted analyses, we found no evidence of an increased rate of breast cancer recurrence associated with the inheritance of at least one Ala allele [adjusted OR = 1.1 (95%CI = 0.65, 2.0) among heterozygotes, and adjusted OR = 0.87 (95%CI = 0.47, 1.6) among homozygotes]. The inheritance of two Ala alleles was also not associated with an increased rate of breast cancer recurrence when compared with wild-type homozygotes and heterozygotes (adjusted OR = 0.81, 95%CI = 0.47, 1.4).

**Table 2:** Observed and expected allelic frequency and Hardy-Weinberg Equilibrium (HWE).

	Wildtype	Heterozygote	Variant
**Observed**	32	44	25
**Expected**	29	50	22
***HWE***	***P = 0.07***		

**Table 3:** Association between MnSOD (*SOD2*) genotype and breast cancer recurrence among women who received cyclophosphamide-epirubicin-5-fluorouracil (CEF) adjuvant chemotherapy; women aged 35 to 69 years at breast cancer diagnosis who resided in Jutland, Denmark, 1991–2001.

	Cases	Controls	Matched* OR (95% CI)	Adjusted§ OR (95% CI)
***SOD2***				
* Wildtype*	35 (30)	66 (32)	1.0	1.0
* Heterozygote*	47 (40)	91 (44)	1.1 (0.67, 2.0)	1.1 (0.65, 2.0)
* Homozygote variant*	35 (30)	52 (25)	0.82 (0.44, 1.5)	0.87 (0.47, 1.6)
Unknown	1 (1)	4 (2)	–	–
***SOD2***				
* Homozygous wildtype & heterozygote*	82 (70)	157 (75)	1.0	1.0
* Homozygous variant*	35 (30)	52 (25)	0.76 (0.45, 1.3)	0.81 (0.47, 1.4)
* Unknown*	1 (1)	4 (2)	–	–

* Conditioned on the matched factors.

§ Adjusted for surgery type, type of adjuvant treatment (i.e., CEF +/− pamidronate, tamoxifen, herceptin) and radiation therapy.

### Meta-analyses

**Figure 2:**
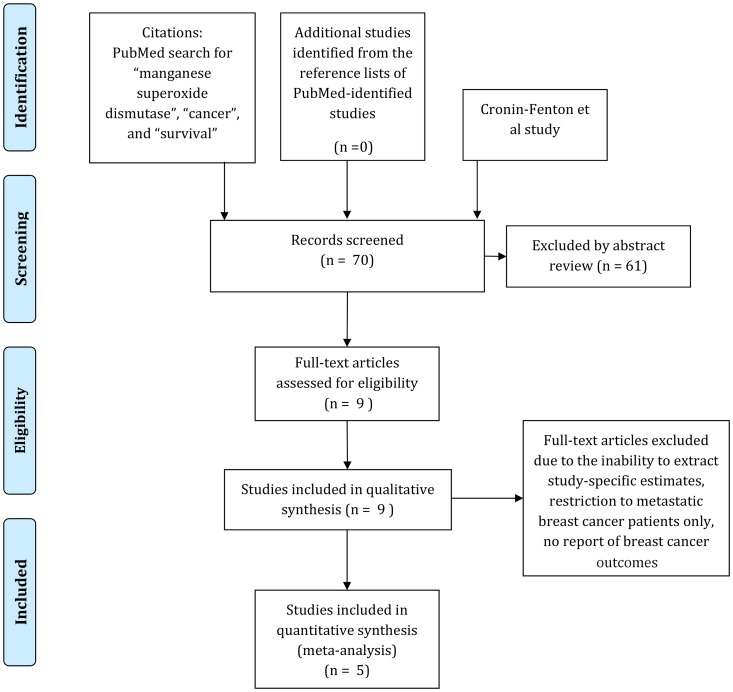
Selection of studies for inclusion in qualitative and quantitative review.

Figure 2 presents a flowchart outlining the process of evaluating articles for inclusion in our meta-analysis. We identified 69 unique records in PubMed based on our primary search terms. Sixty-one of these papers were excluded based on abstract review – most of these studies were based in *in vitro* systems. Nine studies, including our own unpublished work, investigated the association between MnSOD activity and breast cancer outcomes. Two studies were excluded from our meta-analytic models due to the inability to extract study-specific estimates [14], and the investigation of the association of MnSOD protein, rather than genotype, with breast cancer outcome [17]. We also excluded a study by Martin *et al* that investigated *SOD2_102* variant (Genbank AY397775), [16] as the same patients were investigated in the earlier study by Ambrosone *et al*. Therefore all of the studies included in our meta-analysis investigated the *SOD2_47* variant (rs4880). Two studies included between 80–90% Caucasians [11], [13]; the Ji study included 100% Chinese Han patients [15]; the Glynn study included a Norwegian population (100% European descent), and a US population (57% African American, 43% European descent) [10]; and our study is likely to have been 100% patients of European descent (Table S1).

**Figure 3:**
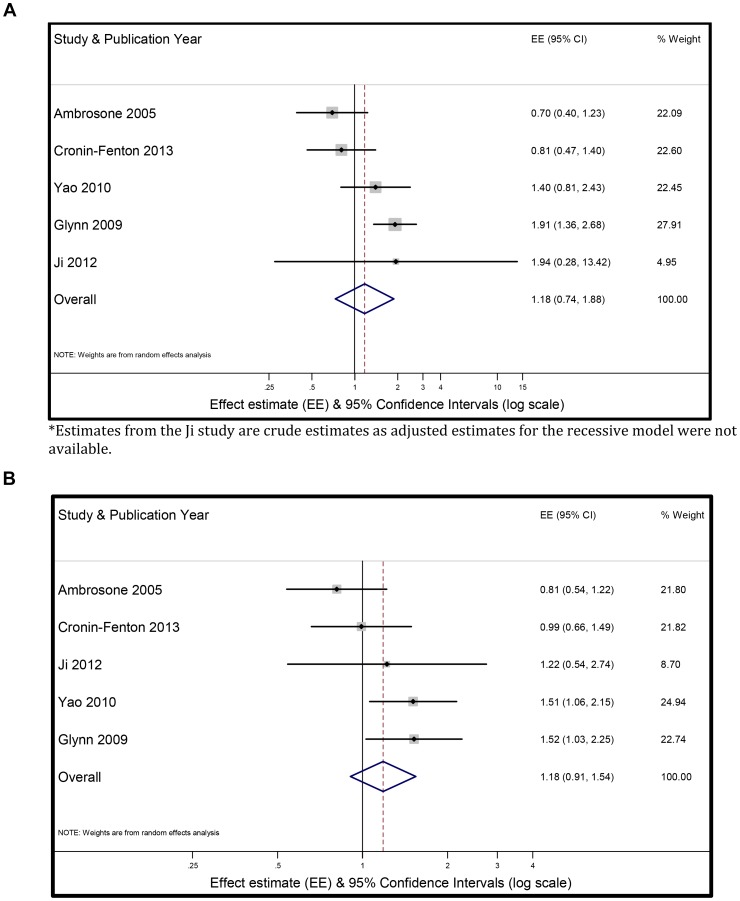
Summary relative effect size (ES) and 95% confidence intervals (95%CI) for the association between inheritance of (3a) two *SOD2* Ala alleles *(recessive model)* versus heterozygote or homozygote wild-type allele and (3b) inheritance of any *SOD2* Ala allele *(dominant model)* (homozygote or heterozygote Ala allele) versus homozygote wild-type (Val/Val) and breast cancer outcome. Summary effect size and 95% CI were estimated using a random-effects meta-analytic model. The size of each square is proportional to the corresponding study’s weight. The horizontal lines represent the confidence intervals. The diamond represents the summary effect size and 95%CI. *.

Figure 3a shows a forest plot of the random-effects meta-analytic model of carriers of two Ala alleles (homozygote variant) of *SOD2* and breast cancer outcomes, compared with carriers of at least one wild-type allele. The summary random-effects estimate was 1.18 (95%CI = 0.74, 1.88). Figure 3b shows the random-effects meta-analytic model of carriers of any Ala allele (heterozygote or homozygote Ala allele) of *SOD2* and breast cancer outcomes, compared with wild-type homozygotes. The summary random-effects estimate was 1.18 (95%CI = 0.91, 1.54). We used the trim and fill method to calculate an adjusted pooled random-effects effect estimate [27]. This method did not add any additional estimates to the funnel plot and the adjusted risk estimate remained unchanged.

## Discussion

In this population-based study, we found no evidence of an association between genetic modification of MnSOD activity and rates of breast cancer recurrence in breast cancer patients treated with CEF adjuvant chemotherapy. This null association persisted even after adjusting for other cancer-directed treatments, including radiation therapy. Our meta-analyses enabled us to contextualize our findings with those in the published literature. They suggest little evidence of an association between a polymorphism in *SOD2* and breast cancer outcomes among women treated with adjuvant chemotherapy.

Several issues should be considered when interpreting our findings. We used the DBCG as a source of comprehensive data on breast cancer patients, tumors, and treatment. The clinical breast cancer database, managed by the DBCG, is considered to be one of the most comprehensive in the world, with exceptionally high data quality [21]. Validation studies of DBCG data have shown that breast cancer recurrence has a positive predictive value of 99.4% [28]. We linked the DBCG records to pathology archives. Thus except for genotyping results, all data and tumor tissues were prospectively collected, minimizing selection bias. As we used risk-set sampling for control selection, the conditional ORs are unbiased estimates of the recurrence rate ratio in the underlying source population [22]. There was little difference between crude (matched) and adjusted analyses. Furthermore, any factor strongly related to breast cancer recurrence is unlikely also to be related to genotype.

There are several plausible explanations for our null results. First, the effect of the *SOD2* variant likely depends on the balance between non-toxic neutralization and toxic ROS generation. We focused specifically on the effect of a polymorphism in an enzyme that acts upstream of other detoxifying enzymes. These other antioxidant enzymes (*e.g.,* myeloperoxidase, catalase) may compensate for a shift in the balance of MnSOD expression in order to maintain cellular homeostasis. A study by Ambrosone *et al.* found that the combined effect of polymorphisms in *SOD2* and *MPO* (the gene encoding myeloperoxidase) was associated with better survival compared with heterozygous or homozygous wild-type carriers of these genes [13]. However, estimates were imprecise given only six exposed cases. Ambrosone *et al.* were also unable to assess the combined effect of polymorphisms in *SOD2* and *CAT*, the gene encoding catalase. Nonetheless, as in our study, they found a near-null association between the *SOD2* polymorphism and the effectiveness of chemotherapy, as measured by breast cancer recurrence (hazard ratio = 0.66, 95% CI = 0.34, 1.29). A null association was also reported in a Chinese study of breast cancer patients [15], in an Italian study focused on MnSOD protein expression and breast cancer survival [17], and in studies evaluating the effect of *SOD2* on survival in patients with other cancers [5].

We included breast cancer patients from a relatively homogeneous population (the Danish population), all of whom had received CEF chemotherapy either alone or combined with endocrine therapy or radiation therapy. This contrasts with some of the other studies, which included patients who received different cancer-directed treatments, across different populations [10], [13], [16]. We note also that our meta-analyses were based on studies that included primarily populations of European descent, and so may not be representative of populations of other racial/ethnic distributions. We were unable to extract an effect estimate from a study in Czech breast cancer patients, which reported an increased risk of recurrence among patients homozygote for the *SOD2* polymorphism [14]. However, they only evaluated the effect of the *SOD2* polymorphism in 30 patients treated with cyclophosphamide. In addition, some of the published studies had very imprecise estimates [10].

We used DNA extracted from tumor tissue rather than non-tumor tissue to assay *SOD2* genotype – similar to the Glynn study [10]. Ideally, we would have used blood samples as a source of DNA, however these were unavailable. An earlier validation study in an overlapping patient population showed excellent genotyping concordance between tumor and non-tumor tissue [29]. Furthermore, a recent paper by Rae *et al* genotyped several CYP2D6 genes in paired tumor and blood and also found good concordance [30]. We note that the paper by Hubackova *et al* [14] reported an increase in *SOD2* transcript levels in tumor compared with paired normal tissue. However, they did not compare *SOD2* genotype in the paired tumor and normal samples. The *SOD2* locus is adjacent to chromosomal regions that are frequently deleted in breast cancer [31], [32]. Although our genotyping results conformed to Hardy-Weinberg equilibrium, we observed a slightly lower proportion of heterozygotes than expected, which may account for the low P-value (0.07). This potential loss of heterozygosity may be an effect of such chromosome six aberrations, and could also have contributed to our null findings.

### Conclusions

In conclusion, we found no association between a functional polymorphism in *SOD2* and breast cancer recurrence. Taken together with the published research, our findings suggest that MnSOD activity, as measured by *SOD2* genotype, is unlikely to be an important factor in predicting response to cyclophosphamide-based chemotherapy.

## Supporting Information

File S1 **Figure A,** Funnel plot showing little evidence of publication bias in studies investigating the association of two *SOD2* polymorphisms with outcomes in breast cancer patients. **Figure B,** Funnel plot showing little evidence of publication bias in studies investigating the association of any *SOD2* polymorphisms with outcomes in breast cancer patients.
(PDF)

Table S1 **Studies included in the qualitative and quantitative review of manganese superoxide dismutase and outcomes in patients with breast cancer.**
(DOCX)
