# Correction: Manganese Superoxide Dismutase and Breast Cancer Recurrence: A Danish Clinical Registry-Based Case-Control Study, and a Meta-Analysis

**DOI:** 10.1371/journal.pone.0092620

**Published:** 2014-03-11

**Authors:** The PLOS ONE Staff

The following information is missing from the Funding section: This study was supported by NIH grants R01CA118708 and R01CA166825.
