# Supplementary material for: Manganese Superoxide Dismutase and Breast Cancer Recurrence: A Danish Clinical Registry-Based Case-Control Study, and a Meta-Analysis
Source: PLoS One. 2014 Jan 31;9(1):e87450. doi: 10.1371/journal.pone.0087450 (PMC3909115; doi:10.1371/journal.pone.0087450)

Figure A: Funnel plot showing little evidence of publication bias in studies investigating the association of two *SOD2* polymorphisms with outcomes in breast cancer patients.

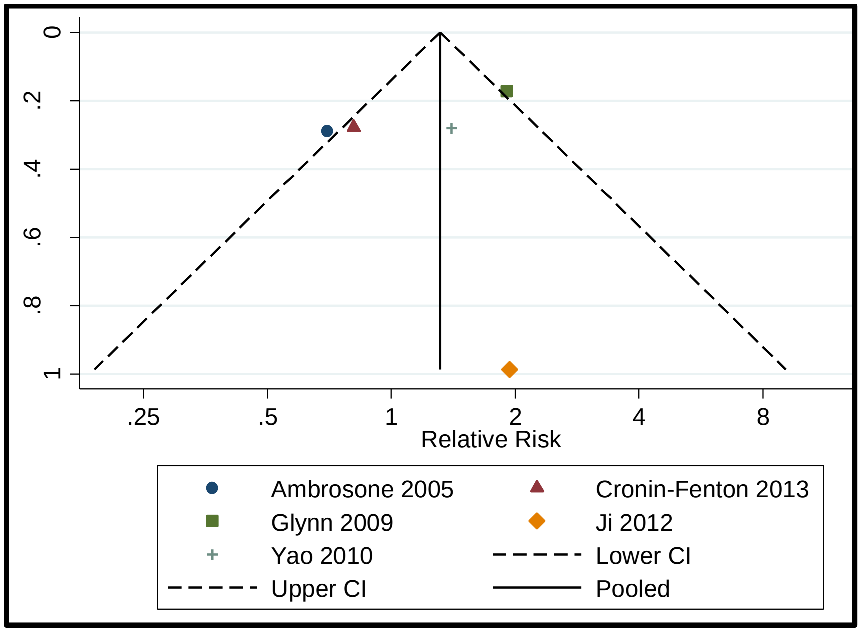

Figure B: Funnel plot showing little evidence of publication bias in studies investigating the association of any *SOD2* polymorphisms with outcomes in breast cancer patients.

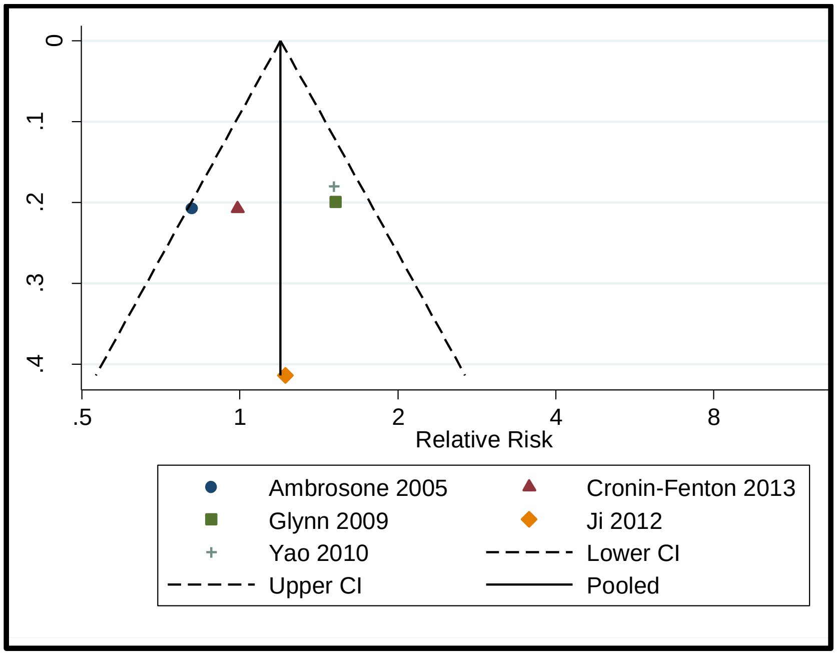

Supplement: File S1 — Figure A, Funnel plot showing little evidence of publication bias in studies investigating the association of two SOD2 polymorphisms with outcomes in breast cancer patients. Figure B, Funnel plot showing little evidence of publication bias in studies investigating the association of any SOD2 polymorphisms with outcomes in breast cancer patients. (PDF) [file pone.0087450.s001.pdf]
