# Supplementary material for: Manganese Superoxide Dismutase and Breast Cancer Recurrence: A Danish Clinical Registry-Based Case-Control Study, and a Meta-Analysis
Source: PLoS One. 2014 Jan 31;9(1):e87450. doi: 10.1371/journal.pone.0087450 (PMC3909115; doi:10.1371/journal.pone.0087450)
Supplement: Table S1 — Studies included in the qualitative and quantitative review of manganese superoxide dismutase and outcomes in patients with breast cancer. (DOCX) [file pone.0087450.s002.docx]

**Table S1: Studies included in the qualitative and quantitative review of manganese superoxide dismutase and outcomes in patients with breast cancer**

| **Reference** | **Date of diagnosis** | **Exclusion criteria** | **Race / Ethnicity** | **Number of outcomes/ Study size** | **Follow-up time** | **Outcomes reported** | **Population source** | **DNA source** |
| --- | --- | --- | --- | --- | --- | --- | --- | --- |
| Ambrosone et al | 1985-1996 | Patients who did not receive adjuvant therapy & patients with missing adjuvant therapy information | 82% Caucasian | 84 deaths/ 279 cases | Median follow-up = 73 months | Overall survival, recurrence-free survival | Hospital-based tumor registry | Paraffin-embedded normal tissue (lymph node, skin or other tissue) |
| Cronin-Fenton et al | 1991-2001 | Patients treated with any other regime besides CEF chemotherapy | Caucasian | 118 recurrent cases/ 213 controls | 2.8 years among recurrent cases; 6.7 years among their matched controls | Breast cancer recurrence | Clinical breast cancer registry | FFPE Tumor tissue |
| Yao et al | 1989-1993 | Women deemed at high risk of recurrence (ER- disease, node positive disease) | 90% Caucasian | 129 recurrences or deaths/458 “treated” patients; 257 recurrences or deaths/874 “untreated” patients* |  | Breast cancer recurrence or death | Post-hoc analyses of clinical trial (South West Oncology Group trial) | FFPE uninvolved lymph nodes |
| Ji et al | 2005-2008 | Patients without measurable lesions or treated with adjuvant chemotherapy | Chinese Han | 52 non-responders (stable/progressive disease) / 159 patients | Median follow-up = 51 months | Non-response to treatment | Clinical series of patients treated at one hospital | Blood samples drawn before neoadjuvant chemotherapy |
| Glynn et al | US-cohort: 1993-2003; Norwegian cohort: various entry points between 1987 and 2001 | US cohort: patients in clinical trials were excluded. | US: 43% Caucasian  Norwegian cohort: 100% Caucasian | US cohort: 83 deaths/ 244.  Norway cohort: 106 deaths/ 329. | Median follow-up = 58 months (US cohort); 65 months Norwegian cohort. | Breast cancer specific survival | US cohort: Patients from surgery lists, covariates and outcome from medical records and pathology reports.  Norway cohort: clinical trials | US cohort: fresh-frozen non-tumor or tumor tissue, buffy coat.  Norway cohort: fresh-frozen breast tumor tissue, buffy coat. |
| Martin et al | 1985-1996 | Patients who did not receive adjuvant therapy & patients with missing adjuvant therapy information | 82% Caucasian | 100 recurrent/ 280 cases  85 deaths/ 291 cases | 73 months? | Overall survival, recurrence-free survival | Hospital-based tumor registry | Archived normal lymph nodes or skin |
| Hubackova et al¤ | 2000-2006 | Patients lost to follow-up, or with stage IV disease | Caucasian? Not reported | Not clear how many recurrences or breast cancer specific deaths in total, but only 30 patients analyzed for *SOD2* and recurrence; 101 patients lost to follow-up; 321 total patients. | Median follow-up = 43 months for patients with family history of breast cancer & 47 months for patients without family history of breast cancer | Breast cancer progression or all-cause mortality | Hospitals | Blood samples (but evaluated *SOD2* in 51 tumor and normal pairs) |
| Bewick et al^§^ | 1992-1997 | Patients with non-metastatic disease | Not reported | 91 breast cancer specific deaths / 95 patients total. | Median follow-up = 10.4 months | Breast cancer specific survival | Hospital clinical series of patients on clinical trials | Cryopreserved, apheresis blood product, peripheral blood or bone marrow |
| Sgambato et al^ | 1994-2001 | Patients who received pre-operative chemotherapy or with family history of breast cancer, or those with missing follow-up information | Caucasian? | 70 breast cancer recurrences; 32 breast cancer deaths/ 134 patients total. | Median follow-up = 72 months | Breast cancer recurrence and death | Hospital-based clinical series | Immuno-histochemistry on FFPE tissue |

*In the Yao et al study, “treated” patients received either cyclosphosphamide-methotrexate-5-fluorouracil or cyclophosphamide-doxorubicin-f-fluorouracil; “untreated” patients did not receive any adjuvant therapy.

¤Hubackova et al study was excluded from meta-analyses due to failure to extract effect estimates and associated 95% confidence intervals.

^§^Bewick et al study was excluded from meta-analyses as it included patients with metastatic breast cancer only.

^Sgambato et al study was excluded from the meta-analytic models as it assessed MnSOD protein expression and not genotype.
